# Effect of GnRH antagonist pretreatment before controlled ovarian stimulation in antagonist protocol for infertile women with PCOS undergoing IVF/ICSI: A propensity score matching analysis

**DOI:** 10.1097/MD.0000000000042965

**Published:** 2025-06-27

**Authors:** Huanying Liang, Jing Zhao, Yan Chi, Jiangchuan Cai, Guifeng Li, Yisheng Zhang, Liling Liu

**Affiliations:** a Graduate School of Guangxi Medical University, Nanning, Guangxi Province, China; b Department of Reproductive Medicine and Genetics Center, The People’s Hospital of Guangxi Zhuang Autonomous Region, Nanning, Guangxi Province, China.

**Keywords:** antagonist protocol, embryo quality, PCOS, pretreatment, PSM

## Abstract

This retrospective cohort study evaluates the clinical effects of an antagonist protocol on in vitro fertilization and intracytoplasmic sperm injection among infertile women with polycystic ovary syndrome. A propensity score matching analysis was conducted of 402 infertile women diagnosed with polycystic ovary syndrome undergoing in vitro fertilization/intracytoplasmic sperm injection cycles. The patients were divided into 2 groups: GnRH antagonist (GnRH-ant) pretreatment protocol (n = 202) and non-pretreatment protocol (n = 200). The primary outcome was the high-quality embryo rate. The metaphase II (MII) oocyte rate was calculated. After adjusting for confounders, the high-quality embryo rate (48.29% vs 42.74%, *P* = .010) was found to be significantly higher in the pretreatment group. However, the number of retrieved oocytes (12.00 vs 12.00, *P* = .878), the MII oocyte rate (76.6% vs 76.0%, *P* = .663), the incidence of ovarian hyperstimulation syndrome (6.82% vs 2.27%, *P* > .05), and the cycle cancelation rate (51.52% vs 51.52%, *P* > .05) were not significantly different between the 2 groups. Similar results were obtained in the propensity score matching analysis of live birth rate (LBR, 46.88% vs 40.63%, *P* = .476). GnRH-ant pretreatment protocol resulted in increased high-quality embryo rates without increasing the cycle cancelation rate and the incidence of ovarian hyperstimulation syndrome. The number of retrieved oocytes, the MII oocyte rate, and the clinical pregnancy outcomes did not differ after GnRH-ant pretreatment.

## 1. Introduction

Gonadotropin-releasing hormone antagonists (GnRH-ants) have been used in assisted reproductive technology (ART) since the 1980s.^[1]^ They have been widely used in in vitro fertilization (IVF) and intracytoplasmic sperm injection (ICSI) cycles because of their advantages of short duration of stimulation, little amount of gonadotropin (Gn), retention of pituitary reactivity, and low incidence of ovarian hyperstimulation syndrome (OHSS).^[2]^

Polycystic ovary syndrome (PCOS) is characterized by hypothalamic–pituitary–ovarian axis dysfunction and metabolic disturbances, such as hyperandrogenemia, hyperinsulinemia, elevated absolute levels of circulating luteinizing hormone (LH) and its relationship to follicle-stimulating hormone (FSH) levels, and chronic anovulation. The 2023 international guidelines highlighted that PCOS diagnosis should integrate Rotterdam criteria with metabolic risk stratification, especially for IVF candidates.^[3]^ The ESHRE Guidelines on ovarian stimulation for IVF/ICSI (2020)^[4]^ were further revised in 2024, emphasizing individualized GnRH antagonist protocols for PCOS patients to balance efficacy and safety.^[5]^ And the latest Expert Consensus on the Standardized Application of Antagonist Protocols in Assisted Reproduction (2022)^[6]^ indicated that high responders were the optimal population for ovulation induction with the antagonist protocol, especially for patients with PCOS. In addition, the antagonist protocol has several shortcomings. The Gn initiation time cannot be arranged flexibly. In addition, the endogenous FSH level was not inhibited because it lacks pituitary downregulation and the transient increase in endogenous FSH during the luteal–follicular transition recruited early antral follicles, thereby affecting the synchronization of follicles. Endometrial receptivity is adversely affected and it eventually influences ART outcomes.^[7,8]^ And high level of LH was reported to affect endometrial receptivity in PCOS women.^[9,10]^ Therefore, reducing the level of LH by optimizing the pretreatment of the antagonist protocol is currently a hot topic in ART.^[11]^

For patients with PCOS, the pretreatment methods prior to controlled ovarian stimulation (COS) involve hormones and nonhormones. The hormonal pretreatment include oestradiol (E_2_), progesterone (P), combined oral contraceptive pills (OCPs), and GnRH-ants. OCP is the most commonly used pretreatment drug for patients with PCOS. OCP pretreatment aims to limit abnormal hormone increases in LH and testosterone. However, this method has been controversial in recent years. In 2019, a meta-analysis^[12]^ included 7 studies to explore the effect of OCP pretreatment before IVF/ICSI on pregnancy outcomes in infertile patients with PCOS and suggested that OCP had an adverse effect on clinical outcomes. A meta-analysis published in 2017^[13]^ showed that P in OCP may have a negative effect on endometrial receptivity. And Wei et al^[14]^ conducted a large multicentre study on the effect of P pretreatment on pregnancy outcomes in patients with PCOS by using an antagonist protocol. The results showed that the pregnancy outcome in the P pretreatment group of patients with PCOS did not significantly improve regardless of whether frozen or fresh embryos were transferred. Confounding factors may have contributed to the results of this retrospective study. Studies^[15]^ found that GnRH-ant pretreatment can reduce the secretion of endogenous FSH and/or LH before Gn initiation and achieve pituitary downregulation, which is similar to the action of gonadotropin-releasing hormone agonist (GnRH-a), possibly improve the clinical outcome of IVF/ICSI cycles. However, some studies suggested no significant difference in the fertilization rate, clinical pregnancy rate (CPR), and sustained pregnancy rate in the GnRH-ant pretreatment group.^[16]^ In 2020, Maryam et al^[17]^ conducted a prospective study to evaluate the effect of GnRH-ant pretreatment on pregnancy outcomes in patients with PCOS treated with an antagonist protocol during the early follicular phase. The results showed that the incidence of OHSS in the GnRH-ant pretreatment group significantly reduced, with more cumulus-oocyte complexes and two pronuclear (2PN) oocytes being produced, and the pregnancy rate was high. However, this study included 88 patients only and the clinical evidence was insufficient. Recent studies in China found that GnRH-ant pretreatment may achieve similar efficacy to OCP pretreatment in patients with PCOS.^[18]^ In conclusion, the characteristics of efficient inhibition and rapid and reversible action on the pituitary gland gave GnRH-ant special advantages as a simple and efficient pretreatment. GnRH-ant pretreatment is expected to be a promising pretreatment protocol to replace OCP or P in high responders, such as patients with PCOS. However, its clinical safety and effectiveness require well-designed clinical research support. At present, few studies focused on this topic and a large space to explore remains.

In conclusion, on the premise of not affecting the outcome of pregnancy, no consensus has been reached on the pretreatment protocol before COS of patients with PCOS with antagonist protocol in IVF/ICSI cycles and no optimal treatment drug has been found to optimize the outcome of COS. This study used retrospective cohort study data and propensity score matching (PSM) method to maximize the balance of baseline data between 2 groups to explore whether GnRH-ant pretreatment could improve the clinical efficacy of IVF/ICSI in patients with PCOS. Some references were provided to patients with PCOS to choose more effective and safer pretreatment methods.

## 2. Methods

### 2.1. Study design and population

This retrospective cohort study included women with PCOS who underwent IVF/ICSI cycles at the Reproductive Medicine and Genetics Center of the People’s Hospital of Guangxi Zhuang Autonomous Region between January 2016 and December 2019. Written informed consent was obtained from all patients before treatment, and they consented to the use of their retrospective data in scientific publications. This study was approved by the Ethics Committee of the People’s Hospital of the Guangxi Zhuang Autonomous Region.

Women with PCOS, who were diagnosed in accordance with the Rotterdam criteria in 2003, underwent IVF/ICSI cycles with a flexible antagonist protocol. Those who did not receive hormonal drug administration 3 months prior to inclusion were enrolled. Data were collected from an electronic medical record system. The exclusion criteria were as follows: uterine lesions (such as adenomyosis, uterine adhesions, and uterine malformations), other diseases that can cause hyperandrogenemia or abnormal ovulation (such as hyperprolactinemia, thyroid diseases, and adrenal tumors); previous surgery on either ovary; dysfunction in the kidney or liver; heart or hematopoietic disease; one of spouse with chromosomal abnormalities; and oocyte freezing retrieval cycle and sperm freezing cycle.

**Figure 1.**
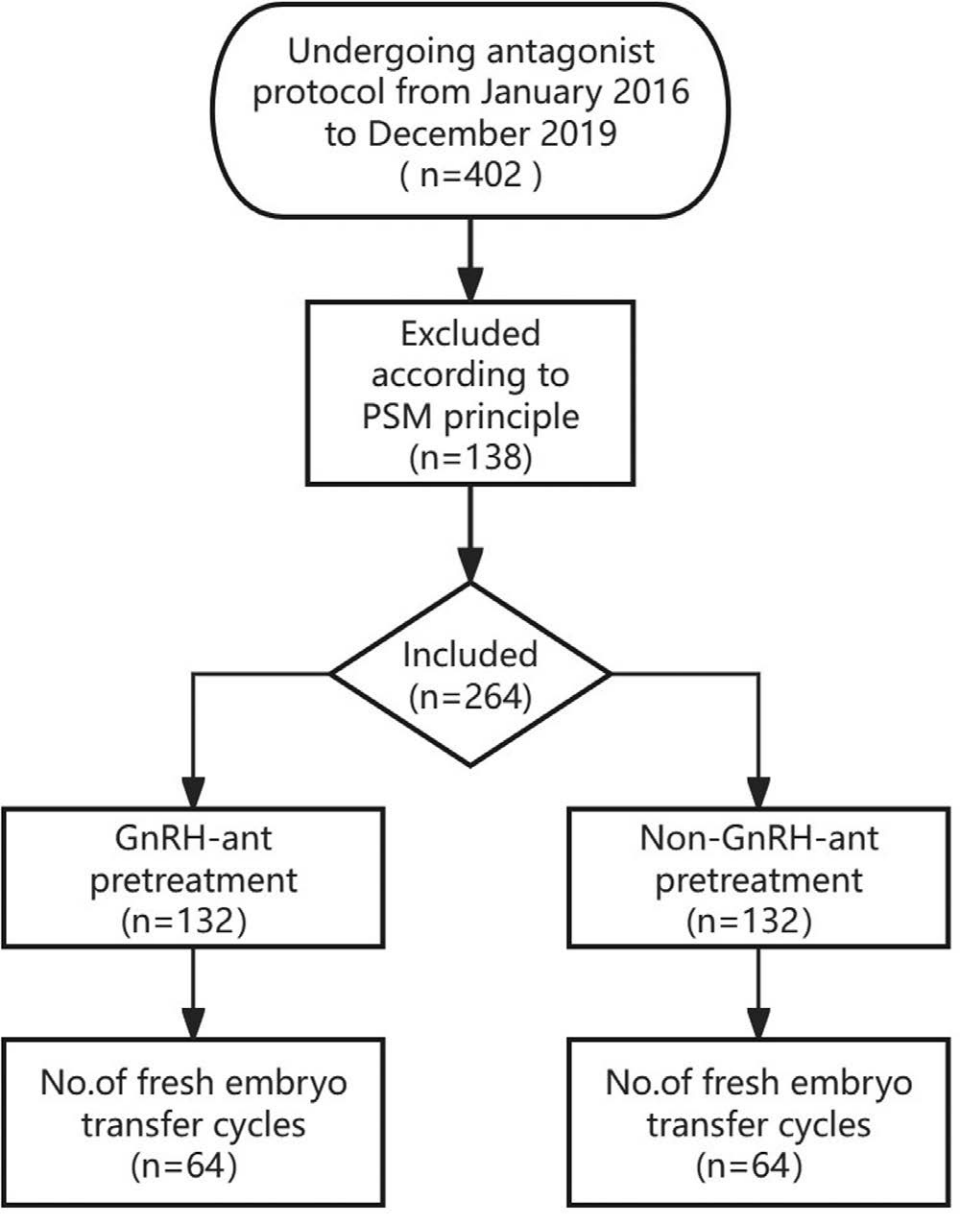
Study flowchart. GnRH-ant = gonadotropin-releasing hormone antagonist, non-GnRH-ant = no gonadotrophin-releasing hormone antagonist prior to ovarian stimulation, PSM = propensity score matching.

In accordance with the use of GnRH-ant pretreatment, the included cases were divided into pretreatment group (n = 202) and non-pretreatment group (n = 200). The basic FSH, LH, E_2_, and P levels were included in the matching variables, and the confounding factors between the 2 groups were controlled by PSM with 1:1 matching. A total of 132 cases were obtained in each group. Amongst them, 64 patients received fresh embryos transferred in each group (Fig. 1).

### 2.2. IVF/ICSI treatment protocols

In the conventional antagonist protocol (non-pretreatment group), ovarian stimulation with Gn was initiated on the second day of the menstrual cycle. In the GnRH-ant pretreatment protocol (pretreatment group), ovarian stimulation was initiated 3 days after GnRH-ant pretreatment (Cetrotide, 0.25 mg cetrorelix acetate, Serono, Inc.), which was administered on the second day of the menstrual cycle. Two types of GnRH-ants were most commonly prescribed: cetrorelix acetate (Pierre Fabre Medicament Production Aquitaine Pharm International Company) and California rick acetate (Zhengda Sunshine Pharmaceutical Group Co., Ltd). During COS, follicle development was monitored by transvaginal ultrasound and the follicle number, follicle size, endometrial thickness, and endometrial type were recorded. Serum FSH, LH, E_2_, and P concentrations were measured and the Gn dosage was adjusted in accordance with hormone and follicle development until follicle maturation. In both protocols, 112.5 to 300 IU of recombinant FSH (Gonal-F, Serono Laboratories Ltd., Geneva, Switzerland) was used for ovarian stimulation. The dose of recombinant FSH was adjusted on the basis of the patient’s ovarian response after ovarian stimulation for 3 to 4 days. In both groups, when serum LH > 10 IU/mL or follicle size ≥ 12 to 14 mm, GnRH-ant was given at 0.25 mg/day until the human chorionic gonadotropin (HCG) trigger day. When the diameter of the 2 dominant follicles was 18 mm or that of 3 dominant follicles was 17 mm, the serum E_2_, LH, and P levels were taken into consideration and intramuscular injection of 5000 to 7500 U HCG (Lizon Pharmaceutical Company) or 250 µg Azer (Merck, Serono, Italy) was selected. Oocytes were retrieved 36 to 38 hours after the triggering.

### 2.3. IVF/ICSI and embryo transfer

Semen analysis and IVF/ICSI preparation were performed on the basis of the World Health Organization Laboratory Manual Human Semen Examination and Treatment (fifth edition). Oocytes were inseminated by incubation with sperms at around (2–3) × 10^5^/mL concentration at 4 hours after oocyte collection in IVF; otherwise, ICSI was performed under a microscope. Fertilization and embryo qualities were checked 12 to 18 hours after fertilization and on day 3, respectively. The embryo quality on day 3 was evaluated in accordance with the number, size, fragmentation, and other indicators of the embryo blastomes as the description of consensus of the laboratory group of the Reproductive Medicine Branch of the Chinese Medical Association and the Istanbul Consensus. Embryo transfer was performed by a specialist physician with a senior professional title under the guidance of an abdominal ultrasound.

**Figure 2.**
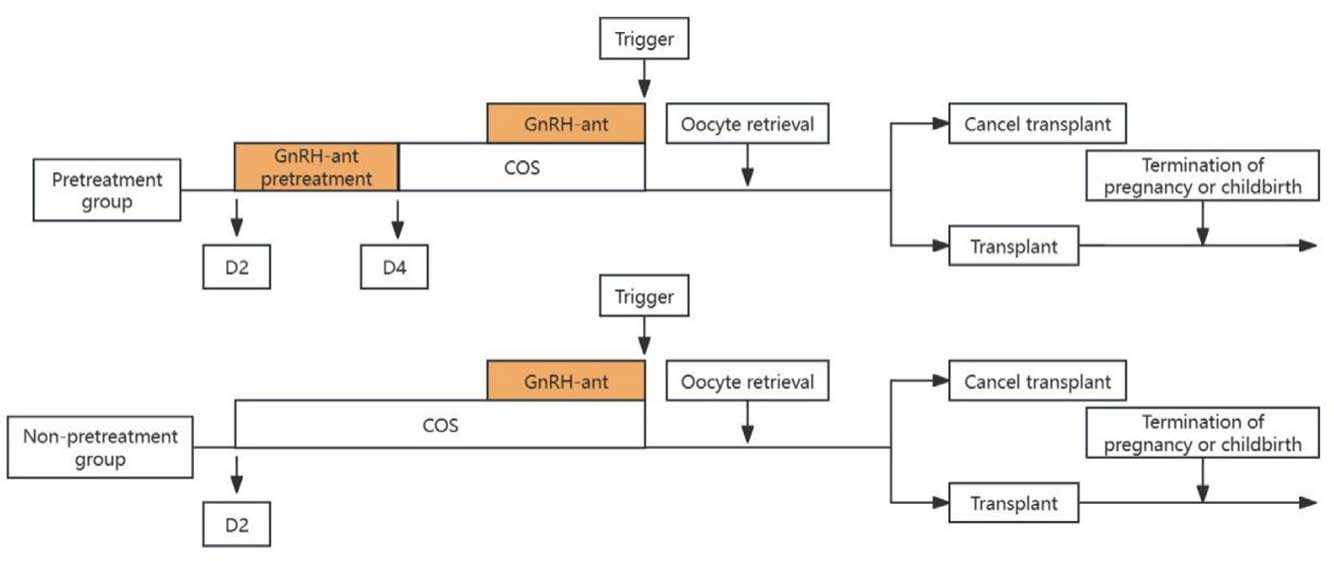
Clinical operation process. COS = controlled ovarian stimulation, D2 = the second day of the menstrual cycle, D4 = the fourth day of the menstrual cycle, GnRH-ant = gonadotropin-releasing hormone antagonist.

For luteal phase support, intramuscular injection of P at 40 mg daily, P vaginal sustained release gel at 90 mg daily or Angelitane at 600 mg daily was administered. Starting from the day of ovulation, if pregnancy was confirmed, the luteal support drugs were gradually reduced to 8 weeks after transplantation and stopped approximately 10 weeks after transplantation. The follow-up nurse recorded the results of the follow-up examinations and reasons for any loss (Fig. 2).

### 2.4. Measure outcomes

The primary outcome was the rate of high-quality embryos defined as grades 1 to 2 with 7 to 9 blastomeres and<20% fragmentation.^[19]^ The secondary outcomes were the number of retrieved oocytes, the metaphase II (MII) oocyte rate, the CPR, the live birth rate (LBR), the cumulative pregnancy rate was the number of patients with positive serum level of beta HCG 25 mIU/ml or above divided by the total number of patients receiving fresh embryo transfer and frozen embryo transfer, and the cumulative live birth rate (CLBR) was the number of deliveries that resulted in at least 1 live birth (defined as the complete expulsion or extraction from a woman of a product of conception after 22 weeks of gestation), per total number of patients receiving fresh embryo transfer and frozen embryo transfer.^[20]^ The incidence of OHSS and cycle cancelation rates were compared between the 2 groups. OHSS was defined using the criteria proposed by Golan and Weissman [2009].

### 2.5. Statistical method

Statistical Package for Social Sciences (IBM, version 26.0) was used for the statistical analysis. Skewed data are presented as the median and inter quartile range. Chi-square test or Fisher exact test (when appropriate) was employed to analyze the difference in frequency data between the 2 groups and *t* test or Mann–Whitney test was used to compare continuous variables. PSM was calculated using the multivariate regression model on the basis of assumed covariates, including base FSH, LH, P, and E_2_, and the caliper value was set at 0.02. The cases were matched at 1:1. The statistically significant variables in the univariate analysis were included in the multifactor negative binomial regression model. The odds ratio (OR) and its 95% confidence interval (CI) were calculated. A probability (*P*) value < .05 indicated that the 2 groups had statistical differences.

## 3. Results

### 3.1. Baseline characteristics

In this study, 202 women with PCOS were included in the pretreatment group and 200 in the non-pretreatment group. After PSM, 132 cases were included in both groups and no statistical significance was found in various baseline characteristics between the 2 groups (*P* > .05) after matching (Table S1, Supplemental Digital Content, https://links.lww.com/MD/P261).

### 3.2. Treatment characteristic in IVF/ICSI

After PSM, the serum LH (4.85 vs 5.34, *P* = .475), FSH (5.81 vs 5.78, *P* = .754), and P (0.30 vs 0.36 *P* = .274) concentrations were similar between the pretreatment and non-pretreatment groups on the initiation day. However, the serum E_2_ concentration on initiation day (33.92 vs 39.00, *P* = .009) was significantly lower in patients with GnRH-ant pretreatment. Those who received GnRH-ant pretreatment had significantly higher levels of LH and P on the antagonist addition day (6.08 vs 4.27, *P* < .001; 0.53 vs 0.40, *P* = .003) but the E_2_ levels (1053.50 vs 1023.00, *P = *.465) were comparable between the 2 groups. On the HCG trigger day, endometrial thickness (10.00 vs 10.00, *P = *.853), E_2_ (2746.00 vs 3101.50, *P = *.064), and P (2.64 vs 1.26, *P = *.028) were not significant. However, LH (2.64 vs 2.26, *P = *.028) was higher in the pretreatment group (Table S2, Supplemental Digital Content, https://links.lww.com/MD/P262). No significant difference was observed in the Gn amount (1200.00 vs 1256.25, *P* = .130) and duration of stimulation (8.00 vs 9.00, *P* = .226) between the 2 groups. Although the total dosage of the antagonist (1.75 vs 1.00, *P < *.001) was higher in the pretreatment group, the GnRH-ant dosage after initiation was less (1.00 vs 1.00, *P = *.001) on initiation day (Table S3, Supplemental Digital Content, https://links.lww.com/MD/P263).

### 3.3. Pregnancy outcomes

**Figure 3.**
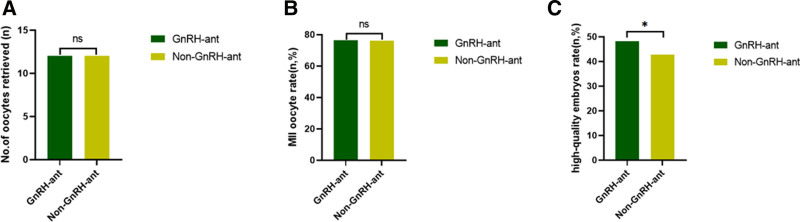
Embryology laboratory outcomes after PSM. (A) No significant difference in No. of oocytes retrieved between the GnRH-ant and non-GnRH-ant groups; (B) no significant difference in MⅡ oocyte rate between the GnRH-ant and non-GnRH-ant groups; (C) the high-quality embryo rate was significantly higher in the GnRH-ant group compared to the non-GnRH-ant group. GnRH-ant = gonadotropin-releasing hormone antagonist. ns = *P* > .05. * *P* < .05.

**Figure 4.**
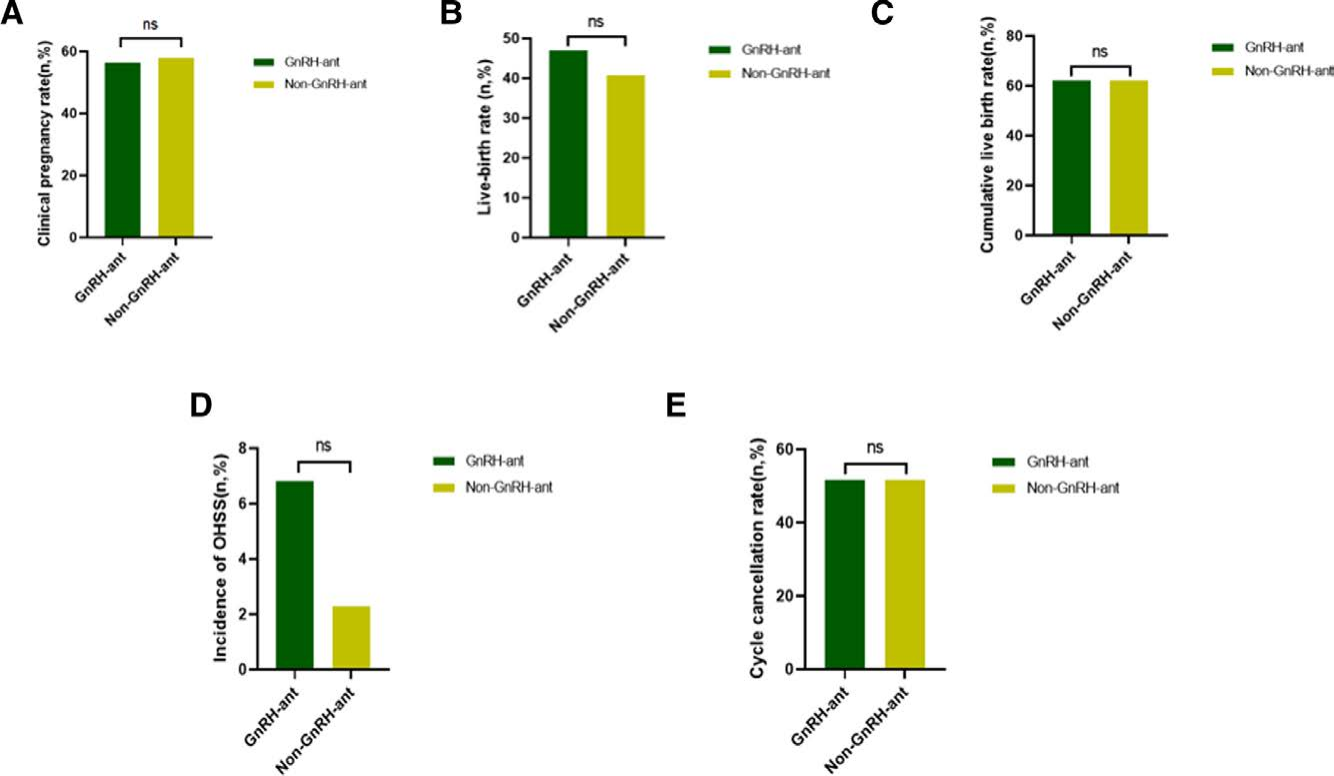
Clinical outcome measures after PSM. (A) No significant difference in clinical pregnancy rate between the GnRH-ant and non-GnRH-ant groups; (B) no significant difference in live birth rate between the GnRH-ant and non-GnRH-ant groups; (C) no significant difference in cumulative live birth rate between the GnRH-ant and non-GnRH-ant groups; (D) no significant difference in incidence of OHSS between the GnRH-ant and non-GnRH-ant groups; (E) no significant difference in cycle cancelation rate between the GnRH-ant and non-GnRH-ant groups. GnRH-ant = gonadotropin-releasing hormone antagonist. ns = *P* > .05.

After PSM, although the number of retrieved oocytes (12.00 vs 12.00, *P* = .878), the transferable blastocyst rate (74.27% vs 72.44%, *P* = .106), the high-quality blastocysts (13.41% vs 15.51%, *P* = 1.425), and the freezable blastocyst rate (46.83% vs 45.13%, *P = *.466) were comparable in both groups, the 2PN rate (83.2% vs 81.3%, *P = *.007) and 2PN cleavage rate (83.4% vs 72.1%, *P < *.001) were significantly higher in patients with GnRH-ant pretreatment. The high-quality embryo rate (48.29% vs 42.74%, *P = *.010) was also significantly higher in the pretreatment group (Table 1, Fig. 3). A total of 92 patients in the GnRH-ant pretreatment group and 99 patients in the non-pretreatment group underwent fresh embryo transfer. After PSM, 64 patients were included in both groups. No significant difference was found in the LBR (46.88% vs 40.63%, *P = *.476), the embryo implantation rate (43.48% vs 40.68%, *P = *.665), the biochemical pregnancy rate (7.81% vs 10.94%, *P = *.581), CPR (56.25% vs 57.81%, *P = *.858), the multiple-birth rate (33.33% vs 25.93%, *P = *.448), and the abortion rate (16.67% vs 29.73%, *P = *.187) between the 2 groups (Table 2, Fig. 4).

**Table 1:** Ovarian stimulation characteristics of the different treatment protocols.

Variables	Before PSM			*P* value	After PSM			*P* value
	GnRH-ant (n = 202)	Non-GnRH-ant (n = 200)	X^*2*^		GnRH-ant (n = 132)	Non-GnRH-ant (n = 132)	X^*2*^	
No. of oocytes retrieved	12.50 (9.00, 18.00)	12.00 (8.00, 20.00)	-	.997	12.00 (9.00, 19.00)	12.00 (8.00, 20.00)	-	.878
2PN egg cleavage	84.17% (1685/2002)	73.88% (1666/2255)	66.98	<.001	83.4% (1079/1293)	72.1% (1060/1469)	50.13	<.001
MII oocyte rate	76.75% (2152/2804)	77.75% (2243/2885)	0.808	.369	76.6% (1404/1831)	76.0% (1421/1868)	0.19	.663
Total fertilization rate			-	-			-	-
IVF	60.94% (1248/2048)	59.69% (1340/2254)	1.806	.179	75.1% (1025/1364)	74.5% (1014/1361)	0.149	.699
ICSI	87.30% (488/559)	81.01% (384/474)	7.704	.006	84.16% (287/341)	82.20% (314/382)	0.874	.350
2PN rate	83.62% (1705/2039)	80.65% (1696/2103)	6.228	.013	83.2% (1092/1312)	81.3% (1080/1328)	7.186	.007
High-quality embryos rate	47.66% (803/1685)	43.40% (723/1666)	6.125	.013	48.29% (521/1079)	42.74% (453/1060)	6.64	.010
Transferable blastocysts rate	72.98% (929/1273)	72.60% (909/1252)	0.289	.591	74.27% (609/820)	72.44% (565/780)	0.106	.745
High-quality blastocysts rate	11.63% (148/1273)	17.25% (216/1252)	16.195	<.001	13.41% (110/820)	15.51% (121/780)	1.425	.233
Freezable blastocysts rate	46.66% (594/1273)	46.09% (577/1252)	0.084	.772	46.83% (384/820)	45.13% (352/780)	0.466	.495

Data are shown as median (Q1, Q3) or percentage of patients (n).

AFC = antral follicle count, AIH = artificial insemination with husband’s semen, BMI = body mass index, E2 = estradiol, FSH = follicle-stimulating hormone, GnRH-ant = gonadotrophin-releasing hormone antagonist, ICSI = intracytoplasmic sperm injection, IVF = in vitro fertilization, LH = luteinizing hormone, non-GnRH-ant = no gonadotrophin-releasing hormone antagonist prior to ovarian stimulation, P = progesterone, PSM = propensity score matching, T = testosterone.

**Table 2:** Pregnancy outcomes between different treatment protocols.

Variable	Before PSM		*X* ^*2*^	*P* value	After PSM		*X* ^*2*^	*P* value
	GnRH-ant (n = 92)	Non-GnRH-ant (n = 99)			GnRH-ant (n = 64)	Non-GnRH-ant (n = 64)		
Live birth rate	43.48% (40/92)	42.42% (42/99)	0.022	.883	46.88% (30/64)	40.63% (26/64)	0.508	.476
Implantation rate	41.21% (68/165)	37.91% (69/182)	0.394	.530	43.48% (50/115)	40.68% (48/118)	0.187	.665
Biochemical pregnancy rate	7.61% (7/92)	13.13% (13/99)	1.551	.213	7.81% (5/64)	10.94% (7/64)	0.305	.581
Clinical pregnancy rate	52.17% (48/92)	54.55% (54/99)	0.108	.743	56.25% (36/64)	57.81% (37/64)	0.032	.858
Multiple-birth rate	37.50% (18/48)	25.92% (14/54)	1.581	.209	33.33% (12/36)	25.93% (14/54)	0.577	.448
Abortion rate	16.67% (8/48)	22.22% (12/54)	0.498	.481	16.67% (6/36)	29.73% (11/37)	1.743	.187

Data are shown as percentage of patients (n).

GnRH-ant = gonadotrophin-releasing hormone antagonist, non-GnRH-ant = no gonadotrophin-releasing hormone antagonist prior to ovarian stimulation, PSM = propensity score matching.

The pretreatment and non-pretreatment groups had 202 and 200 oocyte retrieval cycles, respectively. After PSM, 132 oocyte retrieval cycles were performed in both groups. The pretreatment group had a higher cumulative pregnancy rate (73.48% vs 71.21%, *P = *.680) than the non-pretreatment group. However, no significant differences were found between the groups. Meanwhile, the CLBR (62.12% vs 62.12%, *P *= 1.000) was comparable between the groups (Fig. 4). No significant differences were observed in the incidence of OHSS (6.82% vs 2.27%*, P = *.070) and the cycle cancelation rate (51.52% vs 51.52%, *P *= 1.000) between the 2 groups (Fig. 4).

After PSM was conducted, the high-quality embryo rate was used as the dependent variable. Univariate regression analysis was performed on the potential confounding factors, such as serum LH, E_2_, and P concentrations and the number of follicles with a diameter ≥ 14 mm on HCG day. Then, the covariates of *P* < .100 in the results of univariate analysis were included in the negative binomial multivariate regression model. The results of multivariate regression analysis showed that in the fresh embryo transfer of GnRH-ant pretreatment protocol, the high-quality embryo rate increased with the decrease in serum E_2_ on initiation day (Tables 3 and 4).

**Table 3:** Single-factor regression analysis on high-quality embryos.

Variables	OR	95% CI	*P* value
Age	0.99	0.98–1.01	.416
Duration of infertility	0.98	0.95–1.01	.125
BMI	1.00	0.98–1.02	.713
Basal FSH	1.01	0.96–1.04	.967
Basal E_2_	1.00	0.99–1.00	.033
Basal LH	1.01	0.99–1.02	.384
Basal P	1.19	0.92–1.52	.177
No. of AFC	1.00	0.99–1.01	.874
Gn amount	1.00	0.99–1.00	.278
Stimulation length	0.98	0.96–1.01	.261
Total GnRH-ant amount	1.10	0.96–1.26	.182
GnRH-ant amount after initiating	1.00	0.84–1.19	.988
LH on day of initiating	1.02	0.99–1.04	.136
FSH on day of initiating	0.99	0.96–1.03	.737
P on day of initiating	1.03	0.79–1.34	.823
E_2_ on day of initiating	0.99	0.99–1.00	.004
E_2_ on day of GnRH-ant addition day	1.00	0.98–1.00	.483
LH on day of GnRH-ant addition day	1.01	1.00–1.02	.105
P on day of GnRH-ant addition day	1.17	0.98–1.38	.076
Endometrial thickness on day of hCG trigger	0.96	0.94–0.99	.021
LH on day of hCG trigger	1.00	0.98–1.03	.862
E_2_ on day of hCG trigger	1.00	0.99–1.01	.942
P on day of hCG trigger	1.02	0.88–1.18	.754
No. of follicular ≥ 14mm at trigger day	1.01	0.99–1.02	.397

95% CI = 95% confidence interval, AFC = antral follicle count, BMI = body mass index, E2 = estradiol, FSH = follicle-stimulating hormone, Gn = gonadotropin, GnRH-ant = gonadotrophin-releasing hormone antagonist, hCG = human chorionic gonadotropin, LH = luteinizing hormone, OR = odds ratio, P = progesterone.

**Table 4:** Multifactorial negative binomial regression analysis on high-quality embryos.

Variables	OR	95% CI	*P* value
Basal E_2_	1.00	0.99–1.01	.955
E_2_ on day of initiating	1.03	1.01–1.09	.041
Endometrial thickness on day of hCG trigger	0.97	0.93–1.00	.053
P on day of GnRH-ant addition day	1.12	0.86–1.70	.267

95% CI = 95% confidence interval, E2 = estradiol, GnRH-ant = gonadotrophin-releasing hormone antagonist, hCG = human chorionic gonadotropin, OR = odds ratio, P = progesterone.

## 4. Discussion

This study has the largest sample size, to date, for exploring the clinical effect of GnRH-ant pretreatment on IVF/ICSI in infertile patients with PCOS. The women who received GnRH-ant pretreatment achieved higher high-quality embryo rates and improved oocyte quality, normal fertilization rate and high-quality embryo rate without increasing the cycle cancelation rate nor the incidence of OHSS. However, the number of retrieved oocytes, the MII oocyte rate and the clinical pregnancy outcomes did not differ after GnRH-ant pretreatment.

IVF-ET is the most effective and suitable method to achieve pregnancy when patients with PCOS fail to conceive after first-line life intervention and repeated stimulated ovulation treatment or when combined with pelvic fallopian tube factors, male factors and other infertility factors. The abnormal metabolic function and heterogeneous endocrine disorders of PCOS have a negative effect on oocyte maturation and embryonic development potential, even affecting pregnancy outcomes.^[21]^ Improving the abnormal endocrine status of patients with PCOS before ART improves the clinical efficacy of IVF/ICSI.^[22]^ Meanwhile, patients with PCOS have a high ovarian response and are prone to OHSS during clinical treatment. Effectively improving the success rate and safety of IVF/ICSI in patients with PCOS has always been a hot and challenging issue in the field of human reproduction.

Due to endocrine disorders and other factors, patients with PCOS are prone to premature follicular luteinization and follicular atresia, which lead to difficult pregnancy.^[23]^ The incidence of high serum levels of LH and LH/FSH ratio inversion in patients with PCOS varies from 39.8% to 80%.^[24,25]^ One of the reasons is often attributed to hypothalamus–pituitary–ovary disorder.^[26]^ High LH levels are associated with severe menstrual disorders and increased infertility rate.^[27]^ A large amount of pulse-released GnRH continuously increases serum LH and high levels of LH inhibit the function of FSH. The inverted LH/FSH ratio leads to premature luteinisation of follicles, growth of small sinus follicles, and relatively poor quality oocytes and embryos.^[28,29]^ The key to COS in the antagonist protocol is the control of serum LH levels. In a natural cycle, E_2_ secreted by a single mature follicle reaches the threshold and it is maintained for a certain period of time, thereby promoting LH peak formation through a positive feedback mechanism and the final maturation of oocytes.^[30]^ Emerging evidence from 341 transcriptomic analyses revealed that GnRH-ant pretreatment modulates LH receptor 342 isoforms in granulosa cells, potentially optimizing follicular response to endogenous gonadotropins.^[31]^ However, in patients with PCOS, multi-follicular development is prone to a premature increase in E_2_ levels and the LH peak can occur when follicles are not mature^[32–34]^ during COS. Some studies showed that in the antagonist protocol, when patients with PCOS were added with GnRH-ant after COS initiation, the probability of a premature increase in LH level (26.92%) was higher than that in high responders (15.6%) and the probability of premature ovulation or luteinisation was 2.92%, which occurred in part of large follicles in patients with PCOS. This phenomenon may be attributed to patients with PCOS usually having better ovarian reserves and higher basic serum LH levels, which are more likely to induce multiple follicle maturation, resulting in a rapid increase in E_2_ levels in vivo and stimulation of the positive feedback LH peak.^[35]^ The premature appearance of the LH peak leads to follicular premature ovulation or follicular luteinisation. Lee et al believed that the use of GnRH-ant from the early follicular phase could stabilize the endocrine level of patients with PCOS close to the conditions of normal physiological cycles.^[36]^ Studies showed that the use of GnRH-ant throughout the whole process of COS in patients with PCOS can achieve a satisfactory pregnancy rate,^[37]^ indicating that the use of GnRH-ant in the early follicular phase did not affect the growth of follicles. Animal experiments showed that the use of GnRH-ant in the early stage of the animal estrous cycle (1–5 days) had no effect on cycle length. These results indicated that the use of GnRH-ant in the early follicular phase did not affect the growth and ovulation of dominant follicles.^[22,38]^ Some scholars believe that the use of GnRH-ant throughout the COS tended to improve the clinical outcome by reducing the rapid rise in serum E_2_ concentrations and the risk of early LH peak in patients with PCOS.^[39]^ The present study reached a similar conclusion. The serum LH and FSH levels on Gn initiation day were not significantly different between the 2 groups, whereas the E_2_ level of the pretreatment group on this day was significantly lower than that of the non-pretreatment group (*P* = .009). These results indicate that GnRH-ant pretreatment can delay follicle recruitment and development, reduce serum E_2_ levels before COS and make the endocrine stage before COS close to the normal physiological state in patients with PCOS at the early follicular phase. These results are helpful in preventing the early rapid increase in E_2_ from triggering the early onset of LH peak.

Under physiological conditions, oocyte meiosis begins with a surge of LH in the middle cycle and ends with the formation of mature oocytes before ovulation.^[40]^ Serum LH promotes meiosis in oocytes. Formation of MII oocytes indicates the completion of meiosis and the maturation of oocytes, at which time the oocytes acquire complete developmental ability. The late follicular phase requires appropriate serum LH concentrations to maintain oocyte development and maturation.^[41]^ Serum LH deficiency is likely to occur in the agonist long protocol, with a long maintenance time and deep inhibitory effects on the pituitary gland. COS with an antagonist protocol retained the pituitary reactivity. GnRH-ant inhibited the release of Gn in a dose-dependent manner within a short time, and the synthesis of LH rapidly prevented the premature surge of LH during ovarian stimulation and early ovulation. Inhibition was eliminated 48 hours after drug withdrawal. Therefore, the adverse effects of LH deficiency on oocyte growth and development were avoided. These findings showed that the duration and dosage of GnRH-ant added after COS had certain effects on the concentration of serum LH and oocyte development in the late follicular phase. The study also found that the serum LH concentrations in the pretreatment group on HCG day were higher than those in the non-pretreatment group. This finding is related to the fact that the amount of GnRH-ant in the pretreatment group after Gn initiation was significantly lower than that in the non-pretreatment group, indicating that COS after pretreatment could reduce the amount of GnRH-ant, shorten the duration of GnRH-ant in the late follicular phase and reduce the inhibition of LH. Meanwhile, the 2PN rate (83.23% vs 81.33%, *P = *.007) and 2PN cleavage rate (83.45% vs 72.16%, *P < *.001) in the pretreatment group were higher than those in the non-pretreatment group. These results suggest that pretreatment with GnRH-ant in patients with PCOS led to relatively high levels of serum LH in the late stage of follicles, which had no negative effect on the quality of oocytes but contributed to the eventual development and maturation of oocytes in the late follicular stage. The oocytes obtained were of relatively high quality and had an improved normal fertilization rate, which may be related to the high LH level in late-stage follicles in patients with PCOS. Normal growth and development of follicles require a certain amount of serum LH between the lowest and highest thresholds (the “LH window”). When serum LH is lower than the minimum threshold, insufficient androgen synthesis in theca cells reduces the aromatization of granular estrogen, which results in the eventual development and maturation of oocytes. When LH is higher than the maximum threshold, LH receptors are downregulated, which inhibits granulosa cell proliferation and leads to premature oocyte luteinisation and follicular atresia.^[42,43]^ Studies showed that 1.2 U/L ≤ LH ≤ 5 U/L is the ‘threshold window’ for follicular development and oocyte maturation.^[44]^ For patients with PCOS, whether the ‘LH window’ is the same as that of the normal ovarian responders and whether the threshold window is enlarged or narrowed need further research and discussion. A recent 308 multicenter RCT demonstrated that PCOS patients exhibited broader LH thresholds (0.8–6.2 IU/L) compared to normo-ovulatory controls, suggesting enhanced follicular resilience to LH fluctuations in this population.^[45]^ The results of the present study suggest that in patients with PCOS, the basic serum LH level was generally higher and the serum LH level in the late follicular phase was slightly higher than those of benefited oocyte growth and development for COS with pretreatment. Although the LH level in the pretreatment group was higher than that in the non-pretreatment group on the day of GnRH-ant addition, the increase in the P level did not result in a significant difference between the 2 groups. The results were consistent with those of previous studies.^[46,47]^ In conclusion, the endocrine hormone levels of patients with PCOS were optimized by GnRH-ant pretreatment before COS and the internal environment was in a more normal physiological state. After COS, the dosage of GnRH-ant reduced and the serum LH concentrations in the late follicular phase increased, which was more conducive to the growth and development of oocytes and fertilization.

GnRH-ant rapidly and reversibly inhibits Gn release and early-onset LH peak by competitively binding to the GnRH receptor in the anterior pituitary.^[48]^ The mechanisms of action on follicular development are as follows: (1) follicle recruitment after Gn initiation is increased in the unregulated GnRH-ant protocol to avoid excessive basic serum LH levels. Serum estrogen increases sharply with follicular development, which induces a premature LH peak. Accordingly, the incidence of follicular atresia and follicular luteinisation increases and oocyte quality is compromised.^[49]^ (2) Androgen secretion in theca cells is increased to avoid excessive basic serum LH levels. High levels of serum androgens not only inhibit follicular development but also promote the secretion of estrone, which increases the sensitivity of the pituitary gland to secrete LH; increases the risk of early LH peak; and further affects the oocyte retrieval rate, oocyte quality and endometrial receptivity.^[50]^ High LH levels lead to oocyte apoptosis through premature activation of oocyte meiosis and affect the excretion of the first polar body, which contributes to oocyte chromosome abnormalities and embryo aneuploidy. High LH levels also lead to premature luteinisation of follicular granulosa cells and premature maturation of oocytes by inhibiting the action of inhibin, which influences the development and maturation of oocytes and embryo quality.^[41,51,52]^ The quality of oocytes in patients with PCOS was found to be decreased, mostly because of endocrine abnormalities in PCOS.^[20]^ In the present study, a retrospective analysis was conducted of the clinical data of infertile patients with PCOS who underwent COS using an antagonist protocol. The 2PN and high-quality embryo rates in the pretreatment group significantly increased but no statistical differences were found in the CPR and abortion rates. In the multivariate regression analysis, the E_2_ level on initiation day was an independent factor influencing the high-quality embryo rate in patients with PCOS [OR (95% CI) = 1.03 (1.01–1.09), *P* = .041]. These results suggest that lower E_2_ concentrations in the early follicular phase increased the high-quality embryo rate. Treating with GnRH-ant for 3 days in the early follicular phase (the second day of menstruation), serum LH concentrations were rapidly and reversibly inhibited to prevent the rise in serum E_2_ concentrations by triggering the early LH peak. This pretreatment achieved pituitary downregulation similar to GnRH-a before Gn initiation and mimicked the endocrine environment after the downregulation of the GnRH-a protocol. Meanwhile, the antagonist protocol retains the advantages of pituitary reactivity. The high-quality embryo rate of patients with PCOS pretreated with GnRH-ant significantly increased and the possible reasons were analyzed. The ova of patients with PCOS are susceptible to changes in their follicular fluid microenvironment and the disturbance of the microenvironment possibly affects the normal development of oocytes and normal fertilization.^[53]^ Oocyte quality and fertilization are closely related to embryo quality. The present study showed that GnRH-ant pretreatment optimized the internal environment for follicular development and reduced the adverse effects of long-term endocrine disorders on oocyte quality in patients with PCOS. A relatively normal hormonal state during the process of COS is conducive to the growth and development of oocytes and the improvement of normal fertilization rate and eventually increasing the number of high-quality embryos. The selection of embryos with developmental potential through embryo quality assessment is important to guarantee successful pregnancy outcomes^[54]^ for IVF/ICSI-ET. An increase in the number of high-quality embryos is related to the cumulative pregnancy rate.^[55]^ Recent proteomic studies identified elevated mitochondrial protein expression in 429 oocytes from GnRH-ant pretreated PCOS patients, providing mechanistic insights into improved 430 embryo competence.^[56]^ However, the present study failed to demonstrate that GnRH-ant pretreatment has an effect on pregnancy outcomes between the 2 groups. This phenomenon is possibly related to the fact that only the outcome of fresh embryo transfer after oocyte retrieval was considered and frozen embryo transfer was not included. The total amount of Gn in the pretreatment group was lower than that in the non-pretreatment group, whereas the total amount of GnRH-ant in the pretreatment group increased after 3 days of GnRH-ant pretreatment. A slight difference in total costs was found between the 2 groups. Therefore, pretreatment did not increase the economic burden on the patients. Although different ovulation induction drugs were used in this study, previous studies showed that different GnRH-ants had comparable LBRs and embryo quality during COS.^[57]^

The antagonist protocol was reported to have adverse effects on oocytes and/or endometrial receptivity in high ovarian responders, whose 2PN embryos, high-quality embryo rates and clinical pregnancy rates were lower and cycle cancelation rate was higher^[58,59]^ than those with normal ovarian response. Infertile patients with PCOS showed high pulse LH secretion, LH/FSH ratio imbalance and their receptors unusual level, resulting in decreased expression of endometrial receptivity-related genes during the window period of embryo implantation. The alteration influences asynchronous embryo and endometrial development, reduces endometrial receptivity and eventually leads to high abortion rate and low pregnancy rate after COS.^[60]^ Other studies found that the clinical pregnancy rate of patients with PCOS in the antagonist protocol decreased significantly^[61]^ owing to the poor endometrial receptivity of patients in the antagonist protocol compared with that in the long protocol. Sufficient pituitary downregulation improves endometrial receptivity in patients with PCOS, whose receptivity is poor because of basic endocrine and metabolic disorders.^[62]^ Another study found that the content of HOXA10 in endometrial stromal cells, which is closely related to embryo implantation, was significantly lower in patients treated with the antagonist protocol than in those treated with the long protocol. These results suggest that GnRH-ant has an adverse effect on endometrial receptivity.^[63]^ In the present study, the pretreatment group was treated with GnRH-ant for 3 days in the early follicular phase to achieve pituitary downregulation, by which the disturbed basic endocrine level of patients with PCOS was adjusted and pituitary reactivity was retained. With GnRH-ant pretreatment, the amount of GnRH-ant decreased after Gn initiation. Therefore, the adverse effects on endometrial susceptibility during the induction process decreased. However, no significant difference was found in the endometrial thickness between the 2 groups on the day of HCG administration (*P = *.853). Moreover, no differences were detected in the implantation and clinical pregnancy rates between the 2 groups. Therefore, no negative effect of GnRH-ant pretreatment on endometrial receptivity was observed in this study. Endometrial thickness is an indirect indicator of endometrial receptivity and it was improved by GnRH-ant pretreatment in patients with poor ovarian response.^[64,65]^ For patients with PCOS, the effect of GnRH-ant pretreatment on endometrial receptivity is not clear and further studies are needed.

This study has some advantages despite being a retrospective analysis. Firstly, it had the largest sample size, to date, for exploring the clinical effect of GnRH-ant pretreatment on IVF/ICSI in patients with PCOS. Secondly, this study employed PSM with the principle of pairing on the basis of 264 patients. By using this method, selection bias was reduced to a certain extent and some covariables of the GnRH-ant pretreatment and non-pretreatment groups, which were comparable and did not statistically differ. The conclusion of this retrospective cohort study based on the PSM principle has a certain guiding role for clinical practice due to the few relevant studies and the lack of RCT study with a certain sample size at present.

Although PSM was used, this retrospective cohort study has limitations. Selection bias cannot be avoided because of the incomplete confounding factors considered in the matching and the limitations of the statistical methods. This retrospective cohort study was conducted at a single center and large-sample studies with multiple centers are required in the future.

## 5. Conclusion

In conclusion, GnRH-ant pretreatment of women with PCOS improved the oocyte quality and normal fertilization rate and resulted in a high-quality embryo rate without increasing the cycle cancelation rate and the incidence of OHSS. The number of retrieved oocytes, the MII oocyte rate and the clinical pregnancy outcomes did not differ after GnRH-ant pretreatment. Larger prospective studies are needed to further evaluate the optimal treatment protocol for women with PCOS undergoing IVF/ICSI.

## Acknowledgments

We thank all the staff at the Department of Reproductive Medicine and Genetics Center, the People’s Hospital of Guangxi Zhuang Autonomous Region for their assistance and all participants in the study.

## Author contributions

**Conceptualization:** Huanying Liang, Jing Zhao.

**Data curation:** Huanying Liang, Jing Zhao, Yan Chi, Jiangchuan Cai, Guifeng Li.

**Funding acquisition:** Yisheng Zhang, Liling Liu.

**Supervision:** Yisheng Zhang, Liling Liu.

**Writing – original draft:** Huanying Liang, Jing Zhao.

**Writing – review & editing:** Yisheng Zhang, Liling Liu.

## Supplementary Material


