# Supplementary material for: Effect of GnRH antagonist pretreatment before controlled ovarian stimulation in antagonist protocol for infertile women with PCOS undergoing IVF/ICSI: A propensity score matching analysis
Source: Medicine (Baltimore). 2025 Jun 27;104(26):e42965. doi: 10.1097/MD.0000000000042965 (PMC12212814; doi:10.1097/MD.0000000000042965)
Supplement: Supplementary file 1 [file medi-104-e42965-s001.docx]

| **Supplementary table 1. Baseline characteristics before and after propensity score matching between different treatment protocols.** | | | | | | |
| --- | --- | --- | --- | --- | --- | --- |
| **Variables** | **Before PSM** | | ***P* value** | **After PSM** | | ***P* value** |
|  | **GnRH-ant(n=202)** | **Non-GnRH-ant(n=200)** |  | **GnRH-ant(n=132)** | **Non-GnRH-ant(n=132)** |  |
| Age (years) | 30.00 (27.00, 32.00) | 30.00 (27.00, 32.00) | .847 | 30.00 (27.00, 33.00) | 30.00 (27.00, 32.00) | .723 |
| Duration of infertility (years) | 3.00 (2.00, 5.00) | 3.50 (2.00, 5.00) | .164 | 3.00 (2.00, 5.00) | 3.50 (2.00, 5.00) | .180 |
| BMI (kg/m2） | 22.60 (20.33, 24.97) | 22.22 (19.96, 24.21) | .218 | 23.06 (20.38, 24.95) | 22.10 (19.89, 23.92) | .053 |
| Basal FSH（IU/L） | 5.92 (5.00, 7.14) | 5.58 (4.55, 6.68) | .022 | 5.54 (4.82, 6.64) | 5.76 (4.55, 6.84) | .890 |
| Basal LH（IU/L） | 5.55 (3.84, 7.99) | 6.40 (4.16, 10.06) | .036 | 5.61 (3.82, 8.32) | 6.17 (4.04, 9.26) | .475 |
| Basal E2（ng/L) | 46.00 (35.00, 59.00) | 36.00 (27.82, 47.00) | <.001 | 42.56 (31.99, 52.25) | 39.00 (31.82, 51.08) | .551 |
| Basal P（ug/L) | 0.45 (0.29, 0.61) | 0.22 (0.13, 0.44) | <.001 | 0.40 (0.20, 0.59) | 0.36 (0.20, 0.58) | .214 |
| Basal T（ng/dL) | 49.38(38.38,63.24) | 46.05 (34.18, 60.21) | .100 | 49.71 (37.09, 63.46) | 49.41 (35.42, 62.64) | .795 |
| AFC (n) | 29.00 (26.00, 33.00) | 30.00 (27.00, 35.25) | .056 | 29.00 (27.00, 34.00) | 29.00 (26.00, 33.00) | .757 |
| Infertility factor |  |  | .028 |  |  | .434 |
| Ovulation dysfunction | 11.4% (23/202) | 22.0% (44/200) |  | 11.4% (15/132 ) | 17.4%(23/132 ) |  |
| Pelvic tubal factor | 65.8% (133/202) | 61.0% (122/200) |  | 68.2% (90/132) | 62.1% (82/132) |  |
| Three AIH failure history | 9.9% (20/202) | 6.5% (13/200) |  | 9.8% (13/132) | 7.6% (10/132) |  |
| Male factor | 12.9% (26/202 ) | 10.5% (21/200) |  | 10.6% (14/132) | 12.9% (17/132) |  |
| Protocol fertilization |  |  | .593 |  |  | .775 |
| IVF | 75.2% (152/202) | 78.0% (156/200) |  | 76.5% (101/132) | 74.2% (98/132) |  |
| ICSI | 24.8% (50/202) | 22.0% (44/200) |  | 23.5% (31/132) | 25.8% (34/132) |  |
| Note：Data are shown as median (Q1, Q3) or percentage of patients (n).  GnRH-ant = gonadotrophin-releasing hormone antagonist; Non-GnRH-ant = no gonadotrophin-releasing hormone antagonist prior to ovarian stimulation; PSM= propensity score matching; BMI = body mass index; FSH = follicle-stimulating hormone; LH = luteinizing hormone; E2 = estradiol; P = progesterone; T = testosterone; AFC = antral follicle count; AIH = artifcial insemination with husband’s semen; IVF = in vitro fertilization; ICSI = intracytoplasmic sperm injection. | | | | | | |
