# Supplementary material for: Effect of GnRH antagonist pretreatment before controlled ovarian stimulation in antagonist protocol for infertile women with PCOS undergoing IVF/ICSI: A propensity score matching analysis
Source: Medicine (Baltimore). 2025 Jun 27;104(26):e42965. doi: 10.1097/MD.0000000000042965 (PMC12212814; doi:10.1097/MD.0000000000042965)
Supplement: Supplementary file 2 [file medi-104-e42965-s002.docx]

| **Supplementary table 2. Hormonal changes and endometrial conditions at different time points during COS.** | | | | | | |
| --- | --- | --- | --- | --- | --- | --- |
| **Characteristic** | **Before PSM** | | ***P* value** | **After PSM** | | ***P* value** |
|  | **GnRH-ant (n=202)** | **Non-GnRH-ant (n=200)** |  | **GnRH-ant (n=132)** | **Non-GnRH-ant (n=132)** |  |
| LH on day of initiation (IU/L) | 5.13 (3.40, 7.16) | 5.64 (4.04, 7.75) | .048 | 4.85 (3.35, 7.20) | 5.34 (3.91, 6.82) | .475 |
| FSH on day of initiation (IU/L) | 5.97 (5.16, 7.01) | 5.74 (4.83, 6.80) | .041 | 5.81 (4.91, 6.62) | 5.78 (4.79, 6.80) | .754 |
| P on day of initiation (ug/L) | 0.34 (0.20, 0.55) | 0.22 (0.13, 0.44) | <.001 | 0.30 (0.20, 0.51) | 0.36 (0.20, 0.58) | .274 |
| E2 on day of initiation (ng/L) | 36.74 (28.00, 50.88) | 36.00 (27.82, 47.00) | .420 | 33.92(26.00, 50.03) | 39.00 (31.82, 51.08) | .009 |
| E2 on day of GnRH-ant addition day (ng/L) | 1076.50(794.75,1976.50) | 1000.00(757.00,1719.75) | .042 | 1053.50(760.00,1954.75) | 1023.00(777.00,1801.50) | .465 |
| LH on day of GnRH-ant addition day (IU/L) | 6.18 (3.69, 9.07) | 4.85 (3.12, 7.87) | .002 | 6.08 (3.67, 9.30) | 4.27 (3.02, 6.40) | <.001 |
| P on day of GnRH-ant addition day (ug/L) | 0.58 (0.36, 0.82) | 0.30 (0.20, 0.57) | <.001 | 0.53 (0.30, 0.82) | 0.40 (0.23, 0.60) | .003 |
| Endometrial thickness on day of hCG trigger (mm) | 9.90 (8.50, 11.00) | 10.00 (8.50, 11.03) | .816 | 10.00 (8.50, 11.00) | 10.00 (8.50, 11.17) | .853 |
| E2 on day of hCG trigger (ng/L) | 2928.00(2268.50,3832.00) | 3234.00(2318.50, 4520.25) | .071 | 2746.00(2130.75,3969.00) | 3101.50(2314.00,4248.00) | .064 |
| LH on day of hCG trigger (IU/L) | 2.65 (1.76, 4.23) | 2.51 (1.59, 3.83) | .215 | 2.64 (1.73, 4.09) | 2.26 (1.34, 3.42) | .028 |
| P on day of hCG trigger (ug/L) | 0.70 (0.50, 0.97) | 0.54 (0.38, 0.89) | .001 | 0.70 (0.50, 0.97) | 0.58 (0.40, 0.90) | .073 |
| No. of follicles ≥14mm at trigger day | 11.00 (9.00, 14.75) | 12.00 (9.75, 16.00) | .064 | 11.00 (9.00, 15.00) | 12.00 (9.75, 15.25) | .146 |

Note：Data are shown as median (Q1, Q3).

GnRH-ant = gonadotrophin-releasing hormone antagonist; Non-GnRH-ant = no gonadotrophin-releasing hormone antagonist prior to ovarian stimulation; PSM= propensity score matching; LH = luteinizing hormone; FSH = follicle-stimulating hormone; P = progesterone; E2 = estradiol; T = testosterone; hCG = human chorionic gonadotropin.
