# Supplementary material for: Effect of GnRH antagonist pretreatment before controlled ovarian stimulation in antagonist protocol for infertile women with PCOS undergoing IVF/ICSI: A propensity score matching analysis
Source: Medicine (Baltimore). 2025 Jun 27;104(26):e42965. doi: 10.1097/MD.0000000000042965 (PMC12212814; doi:10.1097/MD.0000000000042965)
Supplement: Supplementary file 3 [file medi-104-e42965-s003.docx]

**Supplementary table 3.** **Hormone characteristics during COS in different treatment protocols.**

| **Characteristic** | **Before PSM** | | ***P* value** | **After PSM** | | ***P* value** |
| --- | --- | --- | --- | --- | --- | --- |
|  | **GnRH-ant(n = 202)** | **Non-GnRH-ant (n= 200)** |  | **GnRH-ant (n = 132)** | **Non-GnRH-ant (n= 132)** |  |
| Gn amount (IU) | 1200.00 (1012.50,1575.00) | 1256.25  (1050.00,1650.00) | .109 | 1200.00 (1003.12,1500.00) | 1256.25 (1050.00,1650.00) | .130 |
| Duration of stimulation (days) | 8.00 (7.00, 10.00) | 9.00 (8.00, 10.00) | .185 | 8.00 (7.00, 10.00) | 9.00 (8.00, 10.00) | .226 |
| GnRH-ant amount after initiating (mg) | 1.00 (0.75, 1.00) | 1.00 (0.75, 1.25) | <.001 | 1.00 (0.75, 1.00) | 1.00 (0.75, 1.25) | <.001 |
| Total GnRH-ant amount (mg) | 1.75 (1.50, 1.75) | 1.00 (0.75, 1.25) | <.001 | 1.75 (1.50, 1.75) | 1.00 (0.75, 1.25) | <.001 |

Note：Data are shown as median (Q1, Q3) .

GnRH-ant = gonadotrophin-releasing hormone antagonist; Non-GnRH-ant = no gonadotrophin-releasing hormone antagonist prior to ovarian stimulation; PSM = propensity score matching; COS = controlled ovarian stimulation; Gn = gonadotropin.
